# Intraoperative Breakage and Endoscopic Retrieval of a Dismantled Triprong Forceps During Ureterorenoscopic Lithotripsy: A Rare Complication and Review of the Literature

**DOI:** 10.7759/cureus.116405

**Published:** 2026-09-17

**Authors:** Deep Jumrani, Ankur Mittal, Vikas K Panwar, Aayush Lakkanna, Shivam Upadhayaya

**Affiliations:** 1 Urology, All India Institute of Medical Sciences Rishikesh, Rishikesh, IND

**Keywords:** biprong forceps, instrument fracture, intraoperative complication, stone retrieval, triprong forceps, ureteric calculus, ureteroscopy, ursl

## Abstract

Ureterorenoscopic lithotripsy (URSL) has become the gold-standard procedure for management of ureteric calculi. Ancillary stone-retrieval instruments such as triprong and biprong forceps facilitate fragment extraction; however, mechanical failure of these devices during active use is a recognized, albeit rarely documented, intraoperative hazard.

We describe a 68-year-old woman who underwent left-sided URSL for a 1 cm upper ureteric calculus at the L4 vertebral level. During stone-fragment retrieval, the distal prongs of a triprong forceps sheared off within the ureter. The retained fragment was successfully retrieved endoscopically under direct vision using a biprong forceps introduced through a larger-calibre ureteroscope, obviating the need for open or laparoscopic exploration.

Instrument fracture during URSL can occur due to metal fatigue from repeated sterilisation cycles, manufacturing defects, or excessive force applied to impacted calculi. Pre-procedural inspection of instruments, avoidance of instrument reuse beyond recommended cycles, complete laser fragmentation of stones before grasping, and availability of rescue instruments (biprong forceps, stone baskets, holmium laser) are paramount preventive and remedial strategies. This case adds to the sparse literature on triprong forceps failure during ureteroscopy and provides a stepwise endoscopic management algorithm for the practising urologist.

## Introduction

Ureterolithiasis constitutes a significant global health burden, with lifetime prevalence approaching 10-15% in industrialised nations and an increasing incidence attributed to dietary, metabolic, and climatic factors. Ureteroscopy with laser lithotripsy (URSL) has evolved as the standard-of-care intervention for ureteric calculi, offering high stone-free rates exceeding 85-95% for upper ureteric stones whilst minimising morbidity compared with open or percutaneous approaches [1-2].

Stone-retrieval accessories--chiefly nitinol baskets, biprong forceps, and triprong forceps--are indispensable adjuncts during URSL. Triprong forceps, characterised by three flexible metallic prongs radiating from a central shaft, are favoured for their firm grasp on irregular calculus fragments [3]. Despite their utility, these instruments are subject to mechanical fatigue, and distal prong fracture during active engagement with a stone can result in a retained foreign body within the ureter--a rare but potentially serious intraoperative complication [4-5].

Published case reports of triprong forceps entrapment or breakage during ureteroscopy are exceptionally sparse. Gite et al. (2016) described two cases of ureteric injury caused by triprong forceps, one involving spontaneous dismantling of the prong assembly while grasping a fragment, and another involving entrapment and subsequent disassembly of the instrument containing the stone. Both were managed by meticulous endoscopic removal of the dismantled parts followed by prolonged DJ stenting [1]. No standardised algorithm exists for the specific scenario of distal prong fracture and intra-ureteric retention in isolation [6]. The present case report documents this complication, its successful endoscopic resolution, and a synthesis of the relevant literature with practical preventive and management guidelines for urologists.

## Case presentation

Patient demographics and preoperative assessment

A 68-year-old woman presented with a two-month history of intermittent left-sided flank pain. Her past medical history was notable for well-controlled hypertension managed with amlodipine; she had no prior urological interventions, coagulopathy, or documented allergy. Physical examination was unremarkable except for mild left costovertebral angle tenderness. Pain was attributed to stone disease only.

Non-contrast computed tomography (NCCT) of the abdomen and pelvis identified a solitary 1 cm radiopaque calculus within the left upper ureter at the level of the fourth lumbar vertebra (L4), with moderate upstream ureteric and pelvicalyceal dilatation (Figure 1). No contralateral pathology was identified. Renal function was within normal limits (serum creatinine 0.9 mg/dL) (Table 1). Urine culture was sterile. The patient was counselled regarding URSL and informed consent was obtained.

**Figure 1:**
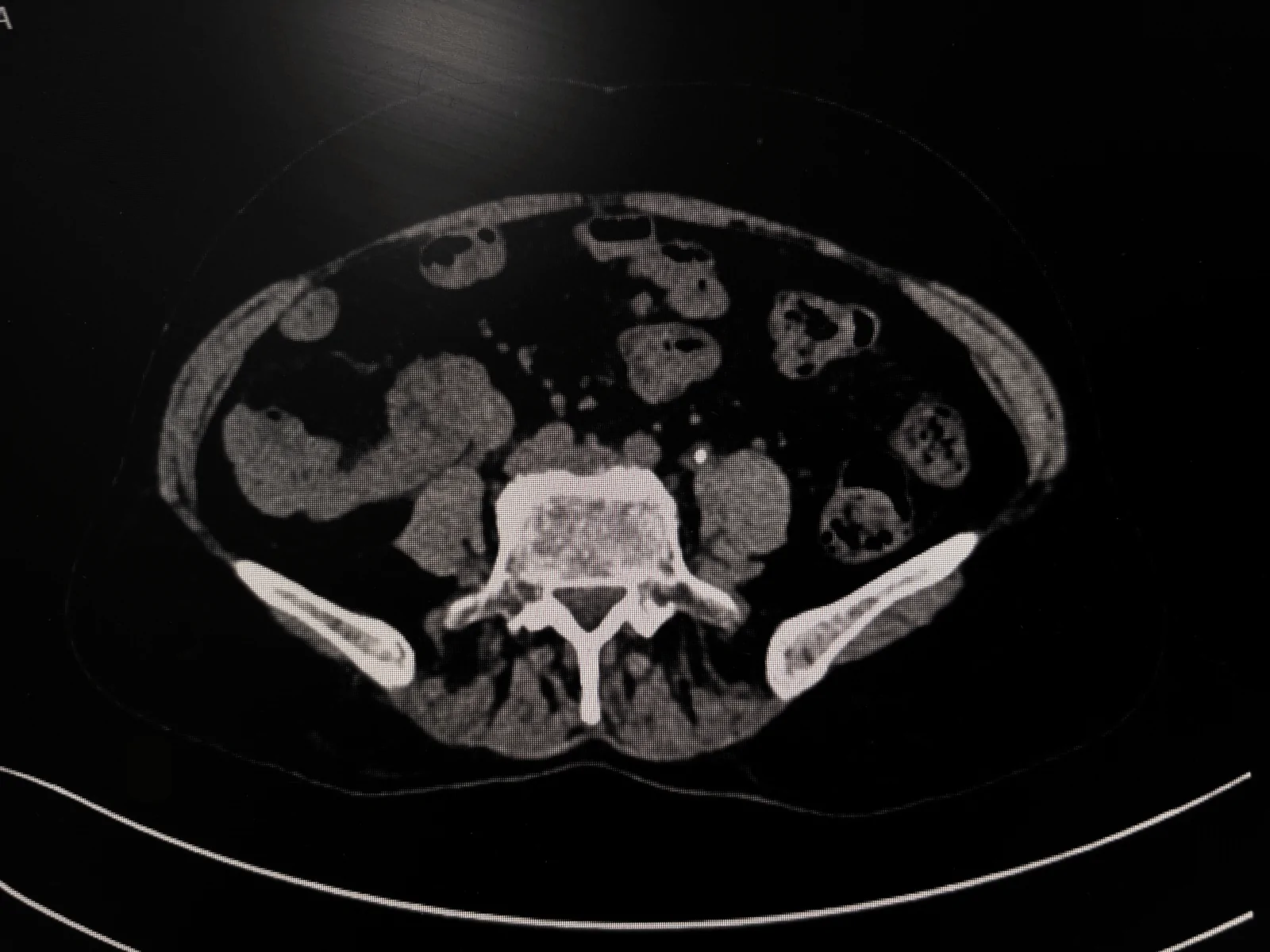
Coronal non-contrast computed tomography image of the kidney-ureter-bladder region showing a 1-cm left ureteric calculus at the L4 level with moderate hydroureteronephrosis.

**Table 1:** Laboratory parameters

Investigation	Patient Value	Unit	Normal Reference Range
Hemoglobin (Hb)	10	g/dL	Male: 13.5-17.5; female: 12.0-15.5
Total leukocyte count (TLC)	7.4	×10³/µL	4.0-11.0
Platelet count	150	×10³/µL	150-450
Blood urea	29	mg/dL	15-40
Serum creatinine	0.9	mg/dL	0.6-1.2
Sodium (Na⁺)	141	mmol/L	135-145
Potassium (K⁺)	3.9	mmol/L	3.5-5.0
Chloride (Cl⁻)	104	mmol/L	98-107
Alkaline phosphatase (ALP)	135	U/L	44-147
Total protein	6.8	g/dL	6.4-8.3
Serum albumin	3.8	g/dL	3.5-5.0
Prothrombin time (PT)	12.5	seconds	11-14
International normalized ratio (INR)	1.1	-	0.8-1.2 (not on anticoagulants)
Activated partial thromboplastin time (aPTT)	32	seconds	25-35
Calcium	9	mg/dL	8.5-10.5
Phosphorus	3.5	mg/dL	2.5-4.5
Magnesium	1.8	mg/dL	1.7-2.2

Intraoperative details

The procedure was performed under spinal anaesthesia with the patient in the dorsal lithotomy position. Rigid cystoscopy confirmed a normal bladder; a 0.035-inch hydrophilic safety guidewire was positioned in the left collecting system under fluoroscopic guidance. A 6/7.5 Fr semi-rigid ureteroscope (Karl Storz, Tuttlingen, Germany) was introduced over the guidewire and advanced to the stone.

The calculus was initially engaged with a triprong grasping forceps for basket-free extraction; however, the fragments proved difficult to grasp in their entirety owing to irregular morphology. During a repeat grasping attempt under direct vision, tactile resistance consistent with instrument binding was noted. Upon withdrawal of the forceps through the ureteroscope working channel, the distal segment--comprising all three prongs and a short portion of the shaft--was found to have detached and remained within the ureteric lumen. The proximal instrument shaft was retrieved intact through the working channel. The patient remained haemodynamically stable, and there was no evidence of ureteric perforation on contemporaneous fluoroscopy.

**Figure 2:**
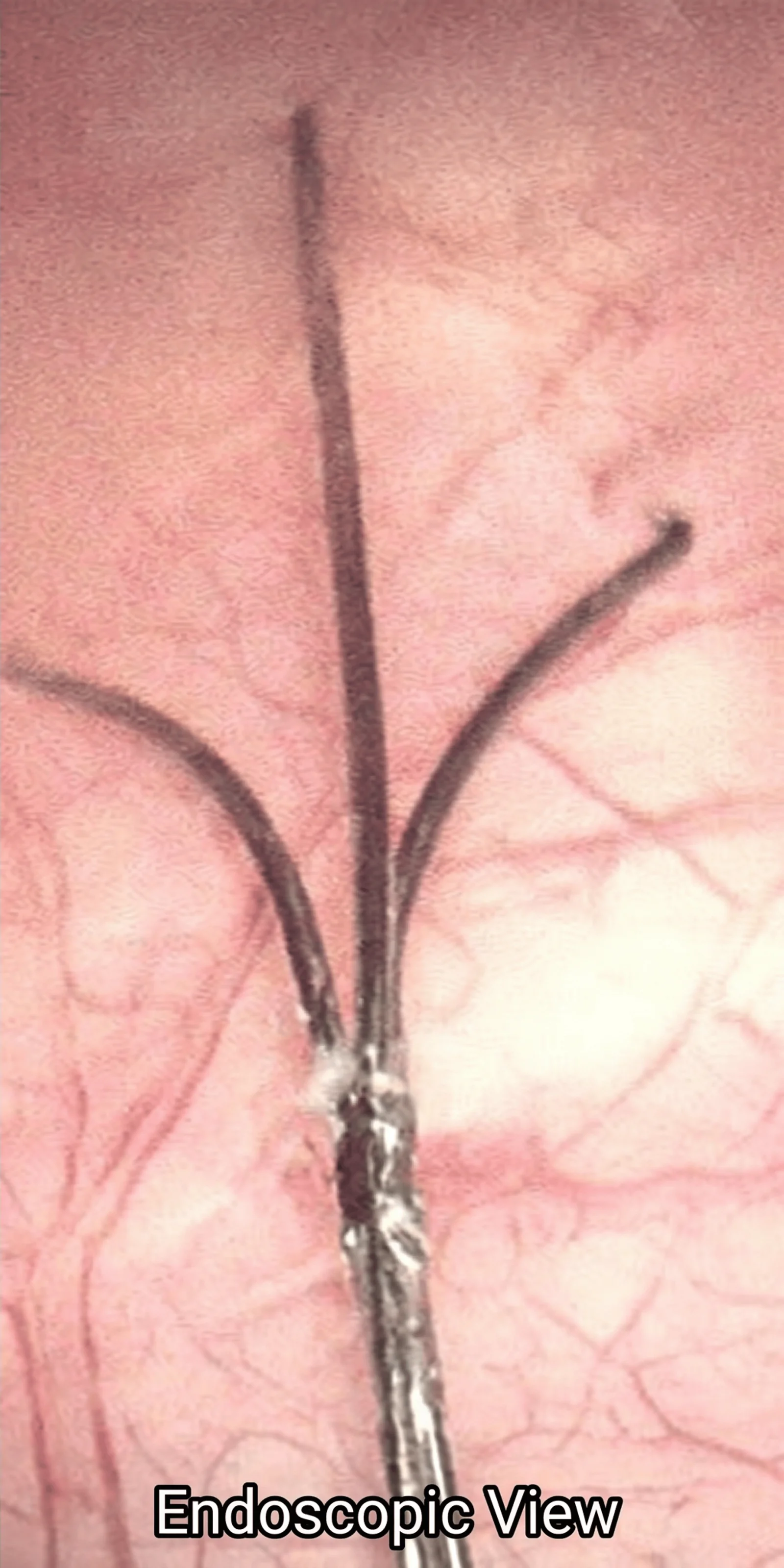
The broken force fragment was visible under direct vision and was present at the same ureteric segment where grasping was attempted.

Management strategy

A decision was made to proceed with immediate endoscopic retrieval rather than abandoning the case and scheduling a second intervention, given the favourable configuration of the retained fragment and the absence of mucosal injury.

The 6/7.5 Fr ureteroscope was exchanged over the safety guidewire for a larger 8/9.5 Fr semi-rigid ureteroscope to accommodate the retrieval instruments required. Under continuous direct ureteroscopic vision, the retained triprong fragment was identified lying along the ureteric axis--a configuration permitting atraumatic manipulation. The residual ureteric calculus was fragmented completely using a holmium:YAG laser (pulse energy 0.6 J; frequency 8 Hz) to reduce the bulk load and create space for instrument manoeuvring.

An attempt was made to grasp the retained metal fragment using a fresh triprong forceps; however, the smooth cylindrical stem of the broken fragment could not be reliably engaged. A biprong forceps was subsequently introduced, and its two parallel jaws were found to provide superior purchase on the metallic stem. The fragment was retrieved intact through the 8/9.5 Fr working channel without resistance or mucosal tearing. Final ureteroscopic inspection confirmed intact ureteric mucosa throughout and no residual stone burden. A 5 Fr × 26 cm double-J (DJ) ureteric stent was placed under fluoroscopic and endoscopic guidance, and the procedure was concluded.

Postoperative course

The patient was discharged on postoperative day one in a stable condition with oral analgesics and an alpha-blocker to facilitate stent tolerance. A kidney-ureter-bladder (KUB) radiograph at discharge confirmed satisfactory stent position and absence of residual calculi. The DJ stent was removed cystoscopically at two weeks in the outpatient setting without complication. Follow-up at six weeks demonstrated complete symptom resolution and normal upper-tract drainage on renal ultrasonography.

## Discussion

The present case makes three distinct contributions. First, it documents intraoperative fracture and distal prong detachment of a triprong forceps during URSL--a scenario distinct from the better-documented phenomenon of forceps entrapment and not previously described as an isolated retrieval challenge [1-3]. Second, it demonstrates successful endoscopic resolution without open surgical conversion, confirming that this complication is amenable to minimally invasive management when appropriate salvage instruments are available. Third, it establishes the superiority of biprong forceps over triprong re-instrumentation for grasping a smooth cylindrical metallic shaft--a technically logical but hitherto undescribed practical observation.

The broader literature on stone-retrieval instrument complications, though still limited specifically to triprong forceps, provides useful context for these observations. Gite et al. described two triprong forceps-related ureteric injuries: spontaneous dismantling of the prong assembly during grasping, and entrapment with subsequent disassembly of the instrument containing the stone; both were managed by meticulous endoscopic removal of the dismantled parts followed by prolonged DJ stenting, without open conversion [1]. De la Rosette et al., synthesising three decades of literature on stone-basketing complications, similarly reported that instrument-related ureteric trauma--ranging from mucosal abrasion to avulsion or intussusception--though infrequent, recurs across device types and is best mitigated through careful instrument selection and awareness of risk factors rather than eliminated outright [2]. In the largest single-centre series available, Geavlete et al. recorded an overall intraoperative complication rate of 3.6% across 2735 semirigid ureteroscopy procedures, with mucosal injury (2.5%) the leading category and iatrogenic instrument-related trauma identified as a distinct indication for further intervention in its own right [3]. Read together, these reports differ from the present case in the specific device and mechanism of failure, but they support the same underlying principle: retrieval-instrument complications during ureteroscopy, while individually uncommon, recur predictably enough across large series to be anticipated rather than treated as anomalies, and endoscopic salvage--rather than open conversion--remains the expected first-line approach.

The decision to proceed with immediate retrieval rather than staging a second procedure was informed by four convergent factors: the fragment was axially oriented and visible under direct vision; there was no evidence of ureteric perforation or mucosal injury; the patient was haemodynamically stable under ongoing anaesthesia; and the requisite salvage instruments were immediately available. Surgeons encountering this complication should systematically weigh these same factors before committing to immediate versus staged retrieval.

The perioperative management of the ureteric mucosa warrants specific discussion. The ureter is a delicate musculomembranous tube with an inner transitional epithelium susceptible to mechanical trauma, with mucosal injury reported in 2.5% of cases in the largest available single-centre ureteroscopy series and stricture formation described as a recognised, if uncommon, sequela of instrument-related ureteric trauma [2,3]; the absence of mucosal injury in our case facilitated a straightforward endoscopic course. Had there been significant mucosal laceration or false-passage creation--either from the initial fracture event or from retrieval attempts--the risk of subsequent ureteric stricture would have been substantially elevated [2], and conservative management with prolonged DJ stenting or early endoscopic surveillance would have been warranted.

The choice of a 5 Fr × 26 cm DJ stent postoperatively was deliberate: the stent served both to ensure ureteric patency in the context of instrumentation-related oedema and as a temporising scaffold should occult mucosal injury manifest as delayed oedema or haematoma formation. Two-week stenting is consistent with contemporary guideline recommendations, which suggest stent durations of approximately one to two weeks, calibrated to procedural complexity and postoperative risk for uncomplicated ureteroscopy with minor ureteric trauma [6].

From a systems-safety perspective, this case illustrates the broader principle of "rescue planning"--the requirement that any endourological surgeon operating with accessory instruments should anticipate instrument failure and ensure the availability of salvage tools before commencing the procedure, consistent with the emphasis placed on instrument selection and preparedness as the primary means of reducing stone-retrieval complications [2]. The unavailability of a biprong forceps, a nitinol basket, or an appropriately-calibred second ureteroscope in the operating theatre would have necessitated procedure abandonment and a second intervention under general anaesthesia.

Management in resource-limited settings: a perspective from India

The present case was managed at a tertiary academic centre in northern India where, as in the majority of public-sector hospitals across the Indian subcontinent, single-use retrieval accessories are not routinely stocked, flexible ureteroscopes are a shared resource, and the range of available salvage instruments is necessarily constrained by procurement budgets. This reliance on reusable equipment is not without a measurable trade-off: a prospective case-control comparison of single-use and reusable flexible ureteroscopes reported scope failure in 7.7% of reusable-scope cases versus 4.4% of single-use cases, a difference that was not statistically significant, indicating that device failure is a quantifiable but broadly comparable risk of reusable-instrument-based practice rather than a marker of substandard care [4]. Despite these limitations, successful endoscopic retrieval was accomplished using equipment universally available at any urological unit equipped for semi-rigid ureteroscopy: a reusable 8/9.5 Fr semi-rigid ureteroscope, a reusable biprong forceps, a holmium:YAG laser, and a standard DJ stent. This outcome underscores that the management of this rare complication is not contingent on sophisticated or expensive technology, but rather on methodical decision-making, maintenance of the safety guidewire, and judicious instrument selection.

For urologists practising in district hospitals or smaller urology units where even a biprong forceps or a second-calibre ureteroscope may not be immediately available, a staged management approach is entirely appropriate and safe. Once instrument fracture is confirmed, the safety guidewire should be secured, a DJ stent placed over the guidewire to maintain ureteric patency and prevent fragment migration, and the procedure abandoned. The patient may then be stabilised and transferred to a centre with the requisite retrieval instruments within 24-48 hours, provided there is no evidence of ureteric perforation or sepsis. The retained fragment, being metallic and non-encrusted in the acute phase, remains endoscopically retrievable during this interval without the risk of encrustation that would accumulate over days to weeks. Early transfer rather than prolonged conservative waiting is strongly recommended, given the risks of encrustation, fragment migration, and secondary mucosal injury that accompany prolonged intraureteric retention of a metallic foreign body.

Where laser lithotripsy is unavailable, as in some district-level facilities in India that rely on pneumatic lithoclast or electrohydraulic lithotripsy (EHL), the principles remain unchanged: do not apply force to disengage the fragment, preserve guidewire access, and decompress the system with a DJ stent in order to buy time. Pneumatic energy should not be directed at the retained metallic fragment, as this risks fragment displacement into the renal pelvis or, rarely, ureteric perforation. If the fragment has migrated proximally into the renal pelvis, a urological referral for percutaneous or flexible ureteroscopic retrieval should be arranged rather than attempting forceful semi-rigid ureteroscopic extraction from below.

In settings where laparoscopy is available but endoscopic retrieval has failed--a situation more likely in smaller centres with limited instrument variety--laparoscopic ureterolithotomy with direct extraction of the metallic fragment is a straightforward and definitive option. Open ureterolithotomy, though rarely required in contemporary practice, remains a valid and dignified last resort that should not be delayed unnecessarily if minimally invasive retrieval is unavailable or has been exhausted. The cultural and medicolegal importance of transparent intraoperative documentation and immediate patient and family counselling regarding the complication cannot be overstated in the Indian medicolegal context; contemporaneous operative notes, fluoroscopy images, and a retrieved-fragment photograph should be secured at the time of management.

## Conclusions

Intraoperative fracture and distal prong detachment of a triprong grasping forceps during URSL is a rare but clinically significant complication that is amenable to endoscopic salvage without open conversion when a safety guidewire is maintained and appropriate rescue instruments--particularly biprong forceps--are available. Successful management depends chiefly on methodical decision-making and pre-emptive rescue planning rather than on sophisticated technology, an outcome with direct relevance to resource-limited practice settings. Prevention remains paramount: mandatory pre-use instrument inspection, adherence to validated use-cycle limits, and complete laser fragmentation of calculi before grasping instrument deployment together minimise the risk of this complication.
